# In-Situ Fabricating V_2_O_5_/TiO_2_-Carbon Heterojunction from Ti_3_C_2_ MXene as Highly Active Visible-Light Photocatalyst

**DOI:** 10.3390/nano12101776

**Published:** 2022-05-23

**Authors:** Wentao Xu, Guoqiang Shu, Shihui Zhang, Lei Song, Kui Ma, Hairong Yue

**Affiliations:** 1 Low-Carbon Technology and Chemical Reaction Engineering Laboratory, School of Chemical Engineering, Sichuan University, Chengdu 610065, China; dexter0315@163.com (W.X.); Shu_SGQ@163.com (G.S.); zsh1234560519@163.com (S.Z.); songlei@scu.edu.cn (L.S.); 2 Institute of New Energy and Low-Carbon Technology, Sichuan University, Chengdu 610207, China

**Keywords:** MXene, heterojunction, degradation, visible light, photocatalysis

## Abstract

Titanium dioxide is a mainstream photocatalyst, but it still confronts great obstacles of poor visible light absorption and rapid recombination rate of photogenerated carriers. Herein, we describe the design of a highly active visible-light photocatalytic system of graphited carbon layers anchored V_2_O_5_/TiO_2_ heterojunctions derived from Ti_3_C_2_ MXene, which demonstrates about 4.58 and 2.79 times higher degradation activity of MB under visible light (λ > 420 nm) than pure V_2_O_5_ and TiO_2_-carbon. Combined with the characterization results, the formed V_2_O_5_/TiO_2_ heterojunction promotes the separation of photogenerated carriers, while the graphitized carbon derived from MXene acts as an electronic reservoir to enhance the absorption of visible light. The ESR results show that superoxide radicals and hydroxyl radicals are the main active species in the reaction system. Therefore, we propose a possible mechanism model to provide a feasible idea for the subsequent design of high-efficiency photocatalysts for environmental treatment.

## 1. Introduction

Currently, the water pollutants such as antibiotic, heavy metal ions and dyestuff in the natural environment system are considered to originate from the rapidly growing industry [1]. In order to cope with the increasingly serious environmental and energy crisis, photocatalytic degradation based on semiconductor materials has become a trending technology with advantages such as mild condition, low energy consumption, and environment-friendly for pollution control [2].

Titanium dioxide (TiO_2_), a mainstream semiconductor material, has been widely considered to be a very developable photocatalyst for photocatalytic degradation, based on its advantages such as high stability, high catalytic efficiency, non-toxicity, and low cost [3]. Nevertheless, the bandgap of TiO_2_ (3.0–3.2 eV) is wide and absorbs only sunlight in the ultraviolet region (only about 5% of solar energy) [4]. In addition, the rapid combination of photogenerated carriers immensely reduces the utilization efficiency of photogenerated carriers [5]. In order to lower the wide band gap and improve the utilization efficiency of visible light, researchers have successfully synthesized a new titanium dioxide photocatalyst by means of element doping including non-noble metal and noble metal element, semiconductor composite, morphological control and so on, which greatly improves the application range of photocatalysts based on TiO_2_ [6]. Among them, semiconductor composites to form heterojunction structure are widely used, i.e., TiO_2_/*g*-C_3_N_4_ [7], TiO_2_@V_2_O_5_ [8], WO_3_/graphene/TiO_2_ [9], TiO_2_@[SO_4_]/CdS [10] and so on. The heterojunction structure formed between semiconductor composites can effectively improve the separation efficiency of photogenerated carriers, thus stimulating efficient photocatalytic performance. Additionally, as a representative narrow-band semiconductor (*E_g_* = 2.3 eV), vanadium pentoxide (V_2_O_5_) has been widely investigated and utilized among the diverse semiconductors driven by visible light. Thus, combining TiO_2_ and V_2_O_5_ to form a heterojunction structure is a feasible way to obtain a promoted visible light-driven photocatalyst [11].

Recently, a new two-dimensional transition metal carbides or nitrides material MXene has been considered an ideal basal for fabrication of TiO_2_-based photocatalyst since its first report by Gogotsi et.al in 2011 [12]. The synthesis of MXene is achieved by selectively extracting A elements (such as Al and Ga) from its parent phase MAX [13]. Its general formula is M_n+1_X_n_T_x_ (*n* = 1, 2, or 3), where M represents early transition metals (such as Ti, V, Nb, and Mo), X represents C and/or N, and T_x_ represents various surface terminals (-OH, -O or -F) [14]. Due to the two-dimensional planar morphology, the excellent electrical conductivity, and the ability to store electrons, MXene has drawn much attention in many fields including photocatalysis [15]. By calcination of Ti contained MXene, TiO_2_ nanoparticles can be in situ grown over the surface to form TiO_2_-Ti_3_C_2_ or TiO_2_-carbon [16], N-doped TiO_2_-carbon [5], and S-doped TiO_2_-carbon [17] with enhanced visible light response than TiO_2_. Among these photocatalysts by in-situ growth of TiO_2_ based on MXene, MXene and carbon layers play the role of substrate and electron reservoir, which leads to enhancing the migration of carriers and light adsorption [18].

Herein, we successfully prepared the graphited carbon layers anchored V_2_O_5_/TiO_2_ photocatalyst derived from MXene by a simple impregnation method, and the photocatalytic activity of MB under visible light irradiation was preliminarily determined. The morphology, chemical state, energy band structure, and optical and electrochemical properties of photocatalyst were characterized by various characterization methods in order to understand its structure and physicochemical properties. According to the above characterization results, we reasonably speculated the mechanism model of the composite catalyst, and explained its reaction mechanism accordingly.

## 2. Materials and Methods

### 2.1. Experimental Reagents

Reagents required for the experiment: ammonium metavanadate, hydrochloric acid, methylene blue and ethanol were obtained from Chengdu Kelong Chemical Co., Ltd. (Chengdu, China), Ti_3_AlC_2_ and LiF were obtained from Shanghai Aladdin Biochemical Technology Co., Ltd. (Shanghai, China). The above reagents are analytical pure without further purification, and deionized water is used in the preparation process.

### 2.2. Synthesis of Ti_3_C_2_

Ti_3_C_2_ MXene defined as TC was synthesized by etching Al from Ti_3_AlC_2_ using mix solution of LiF and HCl. In a typical experiment, 1.0 g of LiF was added to 20 mL of 9 M HCl and stirred for 10 min, then 0.5 g of Ti_3_AlC_2_ was slowly added to the mixed solution and continuously stirred the mixed solution at 35 °C for 24 h [5,19]. The reaction product was thoroughly centrifuged (10 min per cycle at 8500 rpm) and washed with deionized water several times until the pH of the supernatant is neutral, then dried under vacuum at 60 °C overnight [20].

### 2.3. Synthesis of V_2_O_5_/TiO_2_-Carbon

The V_2_O_5_/TiO_2_-carbon heterojunctions photocatalyst was prepared by a simple impregnation method. In a typical experiment, 100 mg Ti_3_C_2_ and a certain amount of ammonium metavanadate (Mass fraction of V element to Ti_3_C_2_ = 10%, 20% and 30%) were added into ceramic crucible containing 5 mL deionized water for ultrasonication 30 min. After that, allow the suspensions to stand for 9 h before drying at 60 °C and then calcined at 550 °C for 4 h with a heating rate of 5 °C/min in air atmosphere. The samples V_2_O_5_/TiO_2_-carbon synthesized with the mass fraction of V element to Ti_3_C_2_ at 10%, 20% and 30% were defined as VOGTO1, VOGTO2, and VOGTO3, respectively. For comparative experiments, the TiO_2_-carbon and pure V_2_O_5_ were prepared by calcining Ti_3_C_2_ and ammonium metavanadate separately, which is defined as GTO and VO, respectively. Moreover, the V_2_O_5_/TiO_2_ was prepared by the same method using TiO_2_ nanoparticles and ammonium metavanadate, which was defined as VTO2.

### 2.4. Catalytic Activity Measurements

The photocatalytic activities of the as-prepared photocatalysts were evaluated by the degradation of MB under visible-light illumination. In a typical photocatalytic experiment, 15 mg of photocatalyst was added to 50 mL of aqueous solution of MB (10 mg/L) and stirred for 60 min under the dark condition to achieve adsorption-desorption equilibrium between photocatalyst and solution. Afterwards, the photocatalytic reaction was performed under the illumination of a 300 W xenon lamp (λ > 420 nm). At given illumination time intervals (15 min), 3 mL of the suspension was taken out and centrifuged to separate the photocatalyst from the reaction system, then the residual concentration of MB was detected by UV-vis spectrophotometer (UV-2355, Unico Instrument Corporation, Shanghai, China).

### 2.5. Catalyst Characterization

The X-ray diffraction (XRD) was tested by an XRD-6100 X-ray diffractometer (Shimadzu Corporation, Kyoto, Japan) with Cu–Ka beam source in 2θ at 5–90° range. Scanning electron microscopy (SEM) was characterized by a REGULUS 8230 (Hitachi, Tokyo, Japan) field emission scanning electron microscopy to observe the morphology of prepared samples. A JEM-F200 (JEOL, Tokyo, Japan) transmission electron microscopy (TEM) was used to gain the TEM and high-resolution TEM (HRTEM) images of photocatalysts. X-ray photoelectron spectroscopy (XPS) was characterized by a K-Alpha (Thermo Scientific, Waltham, MA, USA) photoelectron spectrometer to detect the chemical state of the photocatalysts, and the element binding energy was corrected using the C1s peak at 284.80 eV. The UV-vis diffuse-reflectance spectra (UV-vis DRS) of as-prepared samples was performed with a UV-3600i Plus (Shimadzu Corporation, Kyoto, Japan) spectrometer equipped with an integrating sphere by using BaSO_4_ as reference in the range of 200–800 nm. Photoluminescence (PL) spectroscopy was tested at an excitation wavelength of 255 nm and probed at 400 nm on FLS1000 Spectrometer (Edinburgh Instruments, Edinburgh, UK). Photocurrent measurements were carried out on CHI 660E electrochemical workstation (Chenhua, Shanghai, China). A three-electrode battery made of nanostructured materials on FTO was used as the working electrode, and saturated Ag/AgCl and platinum electrodes were used as the counter electrode and reference electrode, respectively. The electrochemical impedance spectroscopy (EIS) tests were characterized over a 10^−2^ to 10^6^ Hz frequency range. The quasi in situ electron spin resonance (ESR) was performed to detect signals of radicals ⋅O_2_^−^ and ⋅OH spin—trapped by 5,5-dimethyl-l-pyrroline N-oxide (DMPO) in methanol and aqueous solution, respectively, and recorded by a model MS-5000X (Magnettech, Berlin, Germany).

## 3. Results and Discussion

### 3.1. Morphological Analysis

**Figure 1:**
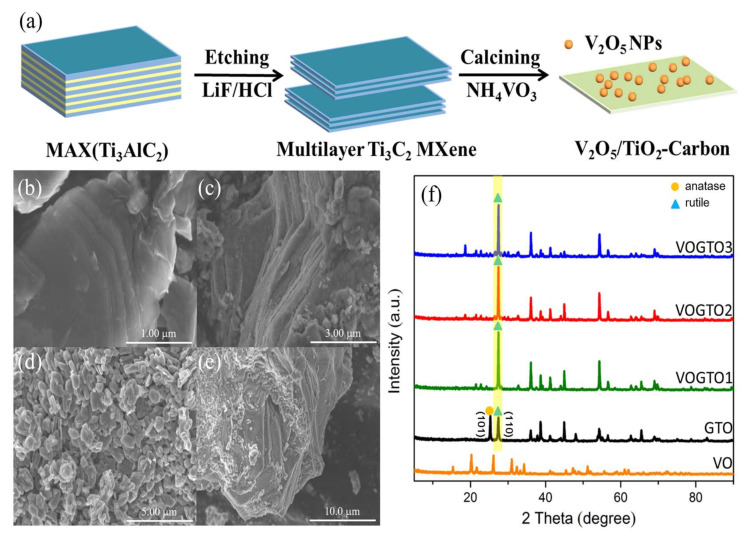
(**a**) Schematic procedure for the synthesis of V_2_O_5_/TiO_2_-Carbon; SEM images of (**b**) TC, (**c**) GTO, (**d**) VO and (**e**) VOGTO2; (**f**) XRD patterns of the photocatalysts.

Typically, a certain amount of multilayer Ti_3_C_2_ MXene (denoted as TC) and NH_4_VO_3_ were mixed and calcined in air to obtain graphitic carbon layers anchored V_2_O_5_/TiO_2_ heterojunctions photocatalyst (Figure 1a). Additionally, the V_2_O_5_/TiO_2_ prepared by the same method using TiO_2_ nanoparticles and ammonium metavanadate was defined as VTO2. The morphological features of TC, GTO, VO, and VOGTO2 were investigated by FE-SEM. The TC represents a typical accordion-like multilayer structure, where the smooth layers and visible jagged edges are separated, suggesting that the Al layer in Ti_3_AlC_2_ was successfully etched by LiF/HCl mix solution (Figure 1b). After calcining TC under 550 °C, GTO was obtained and the surface of the layers get rough and numerous TiO_2_ grow on the surfaces (Figure 1c). Notably, the two-dimensional layered structures are still maintained, suggesting that the oxidation treatment does not destroy the original layer structure, which is mainly due to the C-Ti bonds in Ti_3_C_2_ MXene being easier to break compared with C=C bonds [21]. In addition, the TiO_2_ nanoparticles are uniformly distributed on the surface of the carbon layer, demonstrating that Ti_3_C_2_ MXene is transformed into graphitized carbon layers and TiO_2_ nanoparticles. The pure VO sample shows a nanoparticle-like morphology (Figure 1d). After introducing VO, the samples still show a two-dimensional layered structure, where some aggregates of VO nanoparticles are distributed on the surface of GTO uniformly. The close-packed surface morphology of composites is observed, which is conducive to improving the separation efficiency of photogenerated carriers (Figure 1e).

The crystal phase of as-prepared photocatalysts was investigated by XRD. The Ti_3_C_2_ peak intensities originating from the parent phase Ti_3_AlC_2_ slightly decreased after a mix solution of LiF and HCl treatment (Figure S1). In addition, the (002) peak of Ti_3_AlC_2_ nanosheets is broadened and shifted from 9.4° to a lower 2-Theta angle of 6.1° corresponding to an increase of d-spacing of (002) plane. The low-angle (002) plane of XRD pattern of Ti_3_C_2_ is typical for MXene, which implies a successful preparation of Ti_3_C_2_ MXene [5]. After calcination, the characteristic XRD peaks of Ti_3_C_2_ MXene disappeared entirely, and the characteristic XRD peaks of anatase TiO_2_ (JCPDS No. 89-4921) and rutile TiO_2_ (JCPDS No. 71-0650) were detected (Figure 1f). Furthermore, there is an additional unobvious peak shown at 2θ = 26.23° corresponding to the (002) plane of graphitized carbon, which implies that Ti_3_C_2_ MXene has been converted into TiO_2_ and graphitized carbon after calcination [22]. After introducing V, the TiO_2_ crystalline structure in VOGTO transforms to rutile phase (Figure 1f).

### 3.2. Analysis of Chemical State, Optical and Electrical Properties

**Figure 2:**
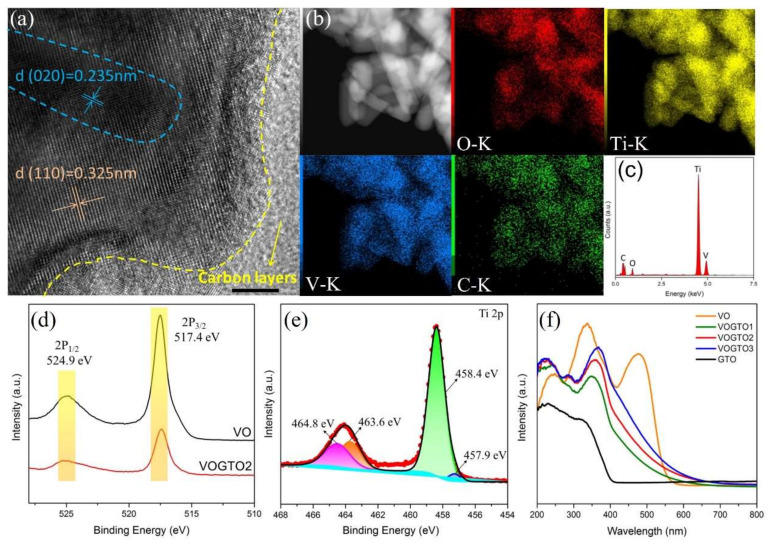
(**a**) HRTEM images of VOGTO2; (**b**) STEM image and element mapping images of VOGTO2; (**c**) EDS spectrum of VOGTO2; XPS spectra of (**d**) V 2*p* of VO and VOGTO2, and (**e**) Ti 2*p* of VOGTO2; (**f**) UV-vis DRS spectra of VO, GTO, VOGTO1, VOGTO2 and VOGTO3.

Figure 2a shows the high-resolution TEM image of VOGTO2. The lattice spacings of 0.235 nm (in blue) and 0.325 nm (in orange) are corresponding to the (020) and (110) planes of the orthorhombic V_2_O_5_ and rutile TiO_2_, respectively (see more details in Figure S2, ESI†). The element signals of C, O, Ti and V are clearly detected and are uniformly distributed on the surface of the VOGTO2 composite in the STEM images (Figure 2b), which combined with EDS results (Figure 2c) further suggests the formation of V_2_O_5_/TiO_2_-carbon heterojunction structure. In order to further understand the chemical state of the photocatalysts, XPS analysis was carried out (Figure S3a, ESI†). Figure 2d shows the V2*p* XPS spectra. The V2*p*_3/2_ and V2*p*_1/2_ peaks at 517.4 eV and 524.9 eV show no shifts in VO and VOGTO2, indicating that the chemical state of V is unchanged. From the high-resolution Ti2*p* spectrum, two peaks of VOGTO2 are observed at 458.4 eV (Ti 2*p*_3/2_) and 464.8 eV (Ti 2*p*_1/2_) in Figure 2e. Furthermore, the Ti 2*p* peaks could be fitted into four peaks at binding energies of around 457.9 eV (Ti^3+^ 2*p*_3/2_), 458.4 eV (Ti^4+^ 2*p*_3/2_), 463.6 eV (Ti^3+^ 2*p*_1/2_) and 464.8 eV (Ti^4+^ 2*p*_1/2_) for both VOGTO2 and GTO (Figure S3b, ESI†) [23]. All the above results imply that the V_2_O_5_/TiO_2_-carbon heterojunction structure was successfully formed.

As the optical properties are one of the essential factors in investigating the photocatalytic mechanism, the UV-vis DRS of prepared photocatalysts was performed. Obviously, the photo-absorption range of GTO extends from the region of ultraviolet light to visible light, the reason of which is that the graphitized carbon could act as the photosensitizer to improve visible-light absorption and adjust conduction band position of TiO_2_ [24]. For VO, it shows the absorption edge at 550 nm, which is attributing to the narrow band gap of VO nanoparticles. In addition, the absorption edge of all VOGTO samples shifts slightly to the visible light region in comparison with the pure GTO, and the absorption intensity in the visible light region increases as the VO amount increases, which is ascribed to the heterojunction structure formation between GTO and VO in the VOGTO samples. The results imply that all these samples can be excited under visible light illumination (Figure 2f). The bandgaps of as-prepared samples are estimated by the Tauc plot, which is derived from the corresponding UV-vis diffuse reflectance spectrum, based on the equation:

$$\alpha h\nu =A{\left(h\nu -E_{g}\right)}^{n/2}$$

where α, h, ν, A and E_g_ rrepresentthe absorption coefficient, planks constant, light frequency, proportionality constant related to the material, and energy band gap, respectively, and the value of *n* is 1 for TiO_2_ and V_2_O_5_ with direct transition semiconductor properties. Thus, from the Tauc plot, the band gap energies of VO and GTO are estimated at around 2.35 eV and 3.20 eV (Figure S4, ESI†), respectively [25]. Notably, the band gap energy of VOGTO composites is between VO and GTO, further demonstrating the formation of heterojunction structure in VOGTO [26].

**Figure 3:**
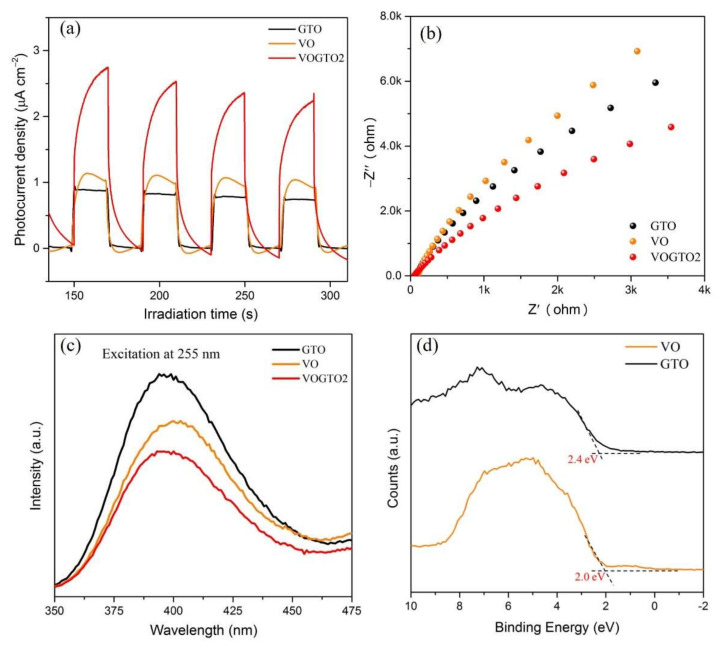
The (**a**) photocurrent density, (**b**) EIS response, (**c**) PL spectra and (**d**) XPS valence band spectra of VO, GTO, and VOGTO2.

The I-t curves display the photocurrent density of prepared photocatalysts coated on FTO as working electrodes under illumination. Compared with GTO and VO photocatalyst, the photocurrent density of VOGTO2 was significantly increased by about three times (Figure 3a), indicating that the separation efficiency of VOGTO2 of photo-generated e^−^-h^+^ pairs has been improved significantly, contributing to the enhanced photocatalytic performance. The lower recombination rate of photogenerated carriers can be attributed to the formation of binary heterojunctions and the effective transfer of carriers on graphite carbon. In addition, by comparing the arc radius of the Nyquist curve recorded on the electrode, EIS can provide a reasonable explanation for the process of effective transfer of photogenerated carriers (Figure 3b). Compared with VO and GTO, the arc radius of the VOGTO2 composite photocatalyst is significantly smaller, which indicates that the close interface between GTO and VO can separate photogenerated carriers faster and more effectively.

To further characterize the charge separation efficiency of prepared samples, photoluminescence (PL) spectra was performed. According to the literature, the intensity of the peak in the photoluminescence emission spectrum (PL) usually originates from the recombination rate of photogenerated carriers, which implies that the greater the intensity of the peak, the faster the recombination rate of photogenerated carriers. Therefore, it is an effective method to evaluate the charge separation efficiency and electron transfer ability of photocatalysts [27]. After the formation of composite photocatalyst between VO and GTO, the photoluminescence (PL) intensity was decreased as compared with pure VO and GTO (Figure 3c), indicating that the recombination rate of the photogenerated carriers in VOGTO photocatalysts is suppressed. Furthermore, in-situ synthesis of V_2_O_5_/TiO_2_/carbon exhibits a strong bonding force, and the photogenerated charges are more easily transferred to the two-dimensional carbon layer to participate in the photocatalytic reaction. Therefore, the visible-light driven photocatalytic activity of V_2_O_5_/TiO_2_/carbon photocatalysts will be higher than that of pure VO and GTO, and this result proved that the synthesis strategy of the photocatalyst is effective. Figure 3d shows the results of XPS valence band spectra. The valence band edge of VO and GTO are 2.0 eV and 2.40 eV, respectively. Combined with the band gap results obtained from UV-Vis diffuse reflectance spectra, the conduction band edge of VO and GTO are −0.35 eV and −0.80 eV, respectively based on the equations of *E_VB_* = *E_CB_* + *E_g_*.

### 3.3. Photocatalytic Performance

The photocatalytic performance of the photocatalysts was evaluated by degradation of MB under visible light (λ > 420 nm) illumination for 90 min.

**Figure 4:**
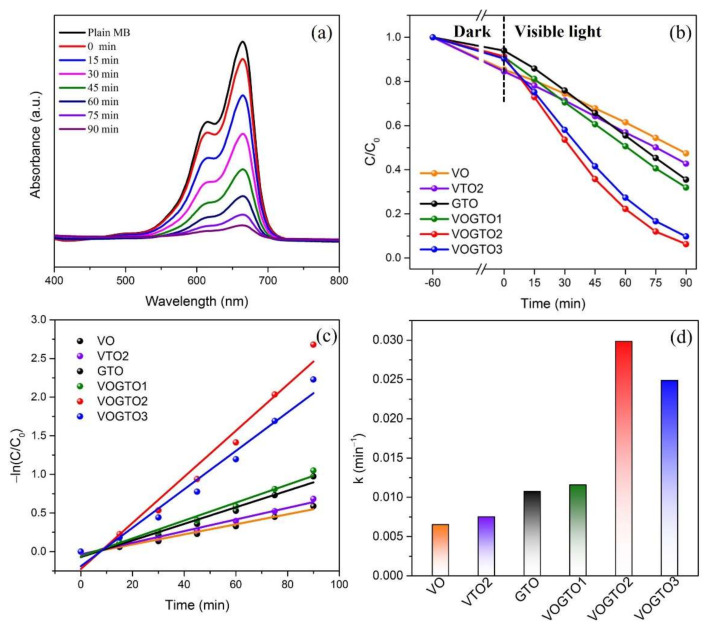
(**a**) Changes of UV−vis spectral absorption (MB) over VOGTO2 under visible-light irradiation; (**b**) Photocatalytic performance of prepared samples for MB degradation under visible-light irradiation; (**c**) Kinetic curves for degradation of MB and (**d**) apparent reaction rate constants over the photocatalysts.

As shown in Figure 4a, the absorption spectra of MB degraded by the VOGTO2 with an increase of the illumination time under visible-light illumination. The intensity of characteristic peaks (664 nm) of MB declined gradually as illumination time went on, suggesting that the MB molecules were gradually degraded after 90 min illumination. The degradation behaviors of MB over VO, VTO2, GTO and VOGTO are shown in Figure 4b. Obviously, the pure VO and GTO show poor photocatalytic activity, the MB degradation rate of which in 90 min was only 53.6% and 64.5%, respectively. Although both VO and GTO can be activated by visible light, the rapid recombination rate of photogenerated carriers greatly limits their photocatalytic activity. As expected, all VOGTO photocatalysts exhibited improved photocatalytic activities. Among them, VOGTO2 exhibited the highest photocatalytic activity after 90 min of visible light illumination. The improved activity can be attributed to the effective separation of photogenerated carriers due to the formation of heterojunction between VO and GTO in the composite photocatalysts. Furthermore, the activity of the VOGTO samples firstly increased with the elevated VO content from 10 wt. % (68.1%) to 20 wt. % (93.8%), and then declined to 90.3% at 30 wt%. The reason is probably that the excessive VO in VOGTO composite photocatalysts will hinder the light absorption of GTO and overlap the active center in GTO, resulting in the decrease of the photocatalytic activity. Therefore, the appropriate VO content plays an essential role in improving photocatalytic activity. It is also worth noting that the content of VO in VTO2 is 20 wt.%, but it still shows poor catalytic activity, which is because there is no graphitized carbon in V_2_O_5_/TiO_2_ to act as the carrier for electron transport and storage to improve photocatalytic activity [28]. Additionally, VOGTO2 also exhibited excellent stability and reusability (Figure S5, ESI†).

Quantitatively, the kinetic behaviors of MB degradation over the samples were also evaluated (Figure 4c,d). The experimental data were fitted by pseudo first-order kinetic model of −ln(*C*/*C*_0_) = *k*t, where *k* represents the rate constant, *C*_0_ is the initial concentration of MB, and *C* is the concentration of MB at time *t* [29]. Based on the plots, the degradation rate constants of the photocatalysts were calculated as 0.65 × 10^−2^, 0.75 × 10^−2^, 1.07 × 10^−2^, 1.15 × 10^−2^, 2.98 × 10^−2^ and 2.48 × 10^−2^ min^−1^ for VO, VTO2, GTO, VOGTO1, VOGTO2 and VOGTO3, respectively. Among them, VOGTO2 exhibited the highest rate constant, which was about 4.58 and 2.79 times higher than that of pure VO and GTO. Generally, high reaction rate constant means high reaction rate and high catalytic activity of the photocatalyst [30]. Besides, we compared our work with previously reported photocatalysts and the results are listed in Table 1. The use of MXene as a precursor of C-doped TiO_2_ and coupled with V_2_O_5_ to prepare the heterojunction photocatalyst is an efficient way to synthesis a photocatalyst with much better visible-light degradation performance.

### 3.4. Analysis of Reaction Mechanism

**Figure 5:**
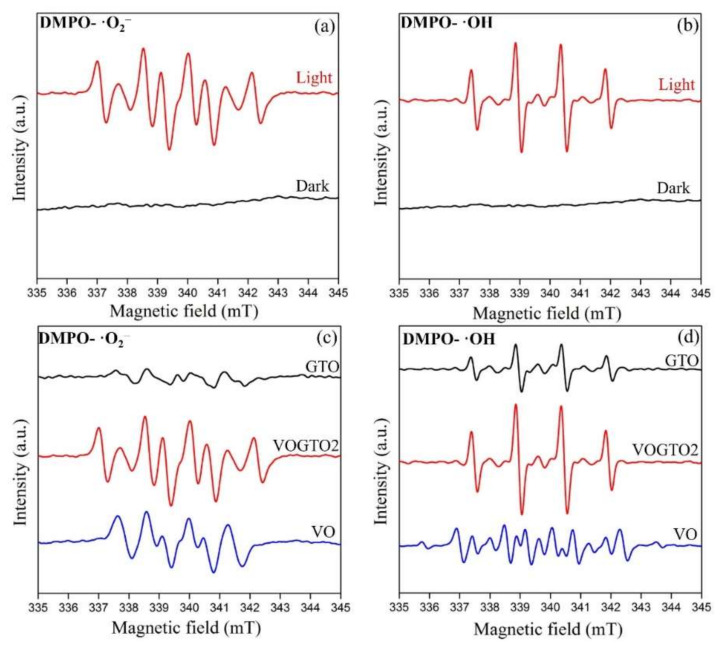
DMPO spin-trapping ESR spectra of VOGTO2 for (**a**) ·O_2_^−^ in methanol dispersion and (**b**) ·OH in water dispersion; The ESR signal intensity of VO, GTO and VOGTO2 for (**c**) O_2_^−^ in methanol dispersion and for (**d**) ·OH in water dispersion.

The mechanism of MB degradation over the V_2_O_5_/TiO_2_-carbon photocatalyst was explored by ESR, in which the DMPO (5,5-dimethyl-1-pyrroline nitrogen oxide) spin trap techniques were applied to detect the free radical species of the degradation system. Figure 5a shows the spectra of DMPO-·O_2_^−^ free radicals. In the dark, there is no ESR signal originating from superoxide radical. After the light is involved, the obvious ESR signal of superoxide radical appears which is due to the reduction of oxygen by photogenerated electrons over VOGTO2 photocatalyst. Similarly, Figure 5b shows the spectra of DMPO-·OH free radicals. There is no ESR signal of hydroxyl radicals in the dark, and the characteristic peak with the intensity ratio of 1:2:2:1 after illumination demonstrates the formation of ·OH free radicals [35]. Intrinsically, the strengthened ESR signal intensity of superoxide radicals and hydroxyl radicals over VOGTO2 is the clue to its better photocatalytic activity (Figure 5c,d) [30]. A comparison of the intensity of the ESR signal measured by the free radicals generated by VO, GTO, and VOGTO2 photocatalyst under the same conditions. VOGTO2 shows the strongest ESR signal intensity of DMPO-·O_2_^−^ radicals and DMPO-·OH radicals compared with VO and GTO. In addition, it can be clearly seen that compared with GTO, the ESR signal intensity of superoxide radical produced by VO is stronger, while that of hydroxyl radical is weaker.

**Figure 6:**
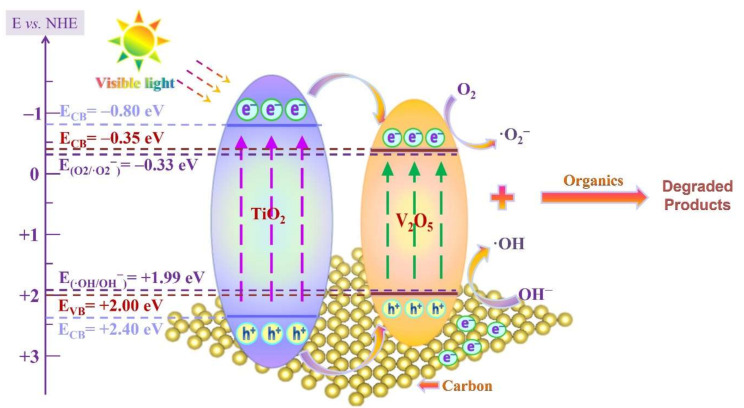
Schematic diagram of photocatalytic degradation of MB by VOGTO2 under visible light irradiation.

Based on the results of XPS valence band spectra and UV-vis DRS, we propose a hypothetical reaction mechanism [7], as shown in Figure 6. In consideration of the photogenerated process of carriers in heterojunction, the GTO of VOGTO2 photocatalyst absorbs photons to arouse photogenerated electrons (e^−^), leaving photogenerated holes (h^+^) on VB under visible light illumination, while the graphited carbon acts as an electronic reservoir to increase the visible light adsorption and accelerate the photogenerated carriers, synergistically boosting the photocatalytic performance [36]. Afterwards, since the conduction band (CB) of VO is more positive than that of GTO, the photogenerated e^−^ on the CB of GTO will transfer to the conduction band of VO and so will the holes [37]. Thus, the photogenerated e^−^ reduce O_2_ to produce superoxide radicals (E^θ^(O_2_/O_2_^−^) = −0.33 V vs. NHE) [38], and the photogenerated h^+^ oxidize OH^−^ to obtain ·OH radicals in water (E^θ^(OH^−^/·OH) = +1.99 V vs. NHE) [38]. Hydroxyl radical and superoxide radical are the main active species for photocatalytic degradation of MB, and the corresponding process can be summarized as follows:

TiO_2_-C + Vis → TiO_2_ (e^−^) + TiO_2_ (h^+^) (1)

TiO_2_ (e^−^) → V_2_O_5_ (e^−^); TiO_2_ (h^+^) → V_2_O_5_ (h^+^) (2)

V_2_O_5_ (e^−^) + O_2_·→·O_2_^−^; V_2_O_5_ (h^+^) + OH^−^ → ·OH (3)

·OH/·O_2_^−^ + MB + Vis → degraded products (4)

## 4. Conclusions

In summary, we have demonstrated a successful and facile design of the graphited carbon layers anchored V_2_O_5_/TiO_2_ photocatalyst derived from MXene, which exhibits excellent photocatalytic activity of MB degradation under visible light illumination. Via tuning the content of V_2_O_5_, the apparent rate constant achieves 2.98 × 10^−2^ min^−1^ over VOGTO2 with 20 wt% V_2_O_5_, about 4.58 and 2.79 times higher than that of pure V_2_O_5_ and TiO_2_-Carbon. Intrinsically, the formed V_2_O_5_/TiO_2_ heterojunction promotes the separation of photogenerated carriers, while the graphitized carbon derived from MXene acts as an electronic reservoir to enhance the absorption of visible light, synergistically boosting the visible-light photocatalytic performance. Our catalyst design and mechanism understanding here can provide guidance for the rational design of a highly active visible-light photocatalytic system.

## Figures and Tables

**Table 1:** Photocatalytic MB degradation of VOGTO2 compared with other reported similar photocatalysts.

Photocatalysts	Amount of Photocatalysts (MB Concentration)	Light Source	Degradation Efficiency	Rate Constant *k* (min^−1^)	References
V_2_O_5_/TiO_2_-SiO_2_	0.5 g/L (10^−^^4^ M)	Visible light	120 min/90%	-	[31]
V_2_O_5_/S-TiO_2_	1 g/L (20 mg/L)	Visible light	240 min/99%	0.018	[32]
V_2_O_5_-TiO_2_/FCC	1 g/L (10 mg/L)	Visible light	120 min/99%	-	[33]
AgVO_3_/V_2_O_5_-TiO_2_	0.5 g/L (20 mg/L)	Sunlight	120 min/95%	0.025	[34]
V_2_O_5_-TiO_2_-C	0.3 g/L (10 mg/L)	Visible light	90 min/95%	0.030	This work

## Data Availability

The data presented in this study are available on request from the corresponding author.

## Supplementary Materials

The following supporting information can be downloaded at: https://www.mdpi.com/article/10.3390/nano12101776/s1. Figure S1: XRD patterns of Ti_3_AlC_2_ and Ti_3_C_2_ MXene, Figure S2: TEM images of (**a**) GTO and (**b**) VOGTO2, Figure S3: XPS spectra of (**a**) survey spectrum sample and (**b**) Ti 2p of GTO, Figure S4: ${\left(\alpha h\nu \right)}^{2}\;\mathrm{versus}\;h\nu$ plot of VO, GTO, VOGTO1, VOGTO2 and VOGTO3, Figure S5: Recycling experiments of VOGTO2 photocatalyst for MB degradation.
