# Supplementary material for: In-Situ Fabricating V2O5/TiO2-Carbon Heterojunction from Ti3C2 MXene as Highly Active Visible-Light Photocatalyst
Source: Nanomaterials (Basel). 2022 May 23;12(10):1776. doi: 10.3390/nano12101776 (PMC9147991; doi:10.3390/nano12101776)
Supplement: Supplementary file 1 [file nanomaterials-12-01776-s001.zip › nanomaterials-1730496-supplementary.pdf]

nanomaterials

Electronic Supplementary Materials

In-Situ Fabricating V_2O_5/TiO_2-Carbon Heterojunction from Ti_3C_2 MXene as Highly Active Visible-Light Photocatalyst

Wentao Xu [1], Guoqiang Shu [1], Shihui Zhang [1], Lei Song [1], Kui Ma [1,*] and Hairong Yue [1,2,*]

[1] Low-Carbon Technology and Chemical Reaction Engineering Laboratory, School of Chemical Engineering, Sichuan University, Chengdu 610065, China; dexter0315@163.com (W.X.); Shu_SGQ@163.com (G.S.); zsh1234560519@163.com (S.Z.); songlei@scu.edu.cn (L.S.)
[2] Institute of New Energy and Low-Carbon Technology, Sichuan University, Chengdu 610207, China
* Correspondence: kuima@scu.edu.cn (K.M.); hryue@scu.edu.cn (H.Y.)

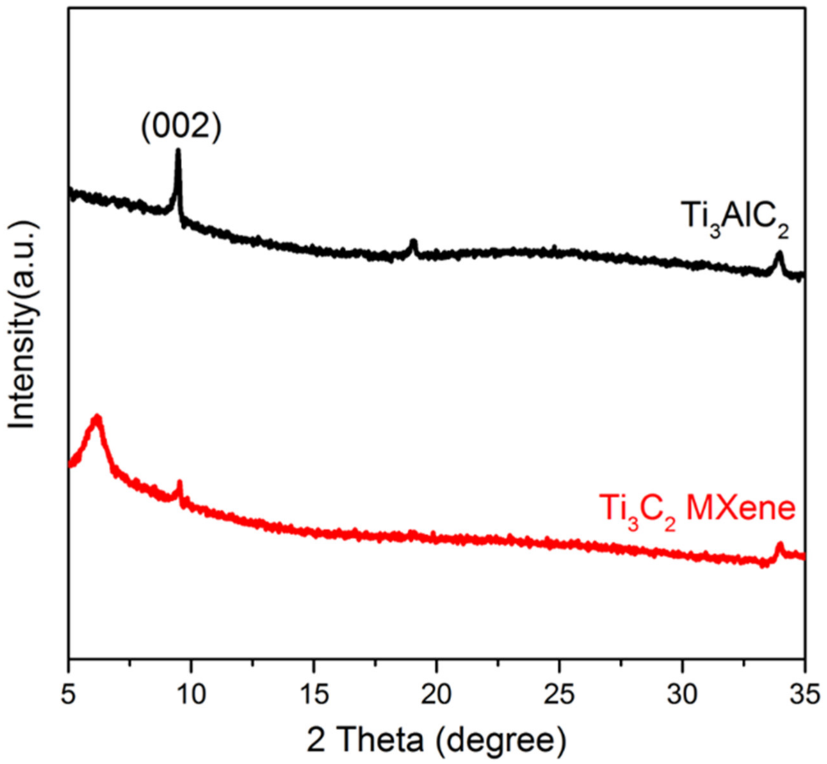

Figure S1. XRD patterns of Ti_3AlC_2 and Ti_3C_2 MXene.

The $Ti_3C_2T_x$ peak intensities originated from the parent phase Ti_3AlC_2 slightly decrease after mix solution of LiF and HCl treatment (Figure S1). In addition, the (002) peak of Ti_3AlC_2 nanosheets is broadened and shifted from 9.4° to a lower 2-Theta angle of 6.1° corresponding to an increase of d-spacing of (002) plane. The low-angle (002) plane of XRD pattern of $Ti_3C_2T_x$ is typical for MXene, which implies that a successful synthesis of $Ti_3C_2T_x$ MXene.

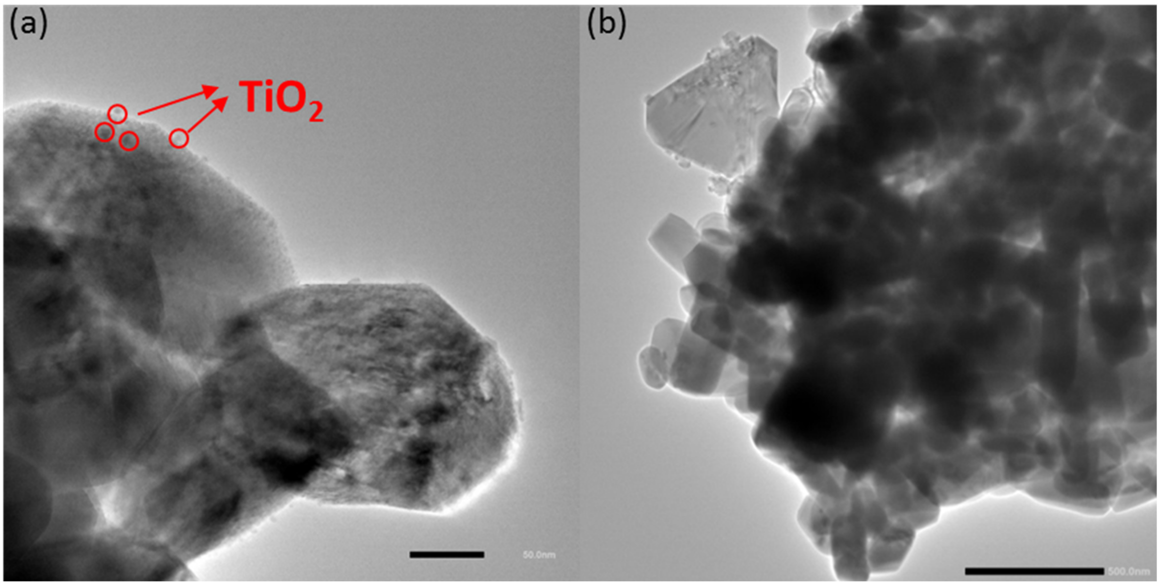

Figure S2. TEM images of (**a**) GTO and (**b**) VOGTO2.

It can be observed that the two-dimensional layered structure is maintained in GTO, which matches with its SEM results. In addition, it is obvious that TiO_2 nanoparticles with nanometer level size are distributed on the surface of graphitized carbon layers (Figure S2a). The introduction of VO leads to solid agglomeration, and a tightly packed surface morphology is formed (Figure S2b), which is in good agreement with the SEM results.

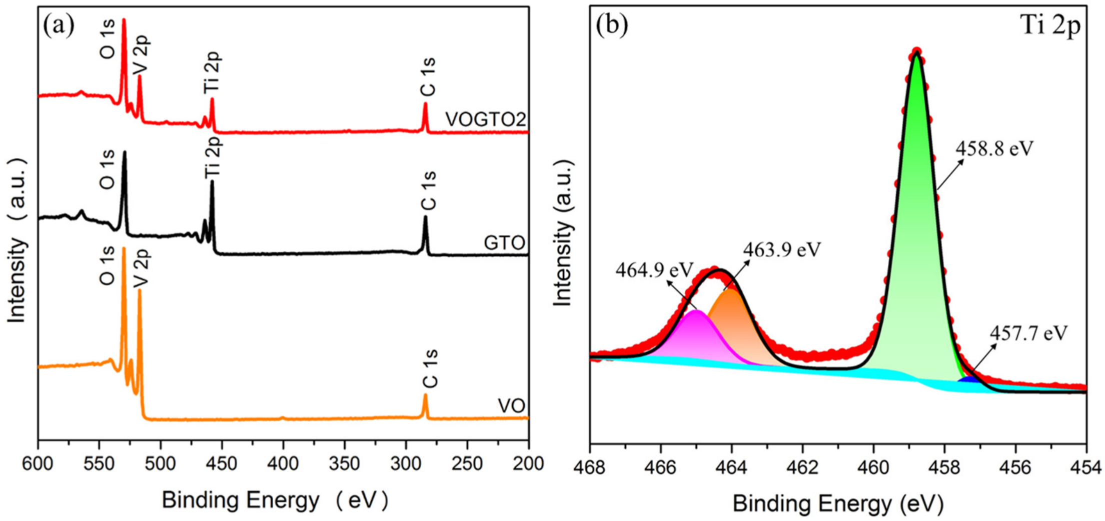

Figure S3. XPS spectra of (**a**) survey spectrum sample and (**b**) Ti 2p of GTO.

All the elements of TiO_2, V_2O_5 and grapheme can be found in the composite VOGTO2, in which the intensity of Ti 2p decreases and the intensity of O 1s increases comparing with GTO sample, due to the introduction of VO (Figure S3a).

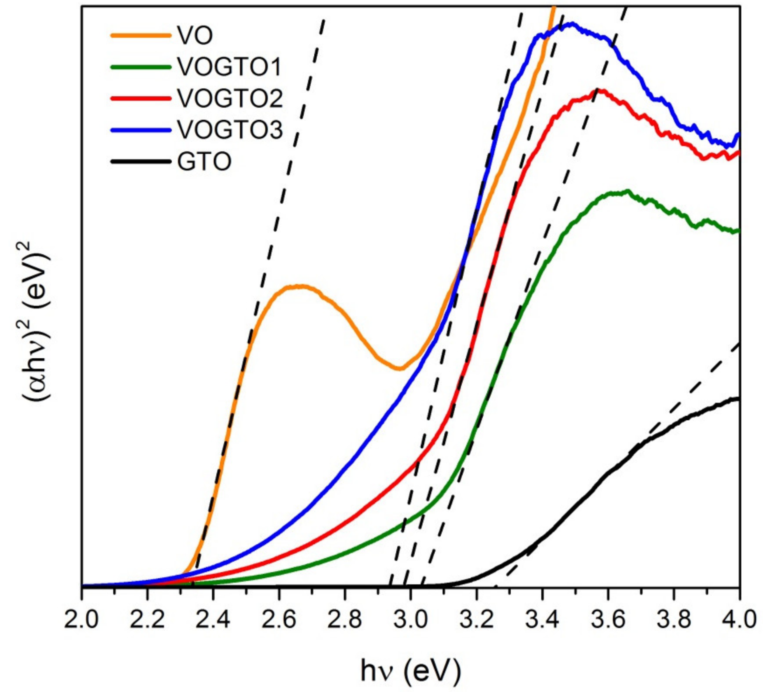

Figure S4. $(\alpha h\nu)^2$ versus hν plot of VO, GTO, VOGTO1, VOGTO2 and VOGTO3.

The band gap energies (Figure S4) of as-prepared samples can be calculated by the the Tauc plot based on the equation:

$$\alpha h\nu = A\left(h\nu - E_g\right)^{n/2}$$

where α, h, ν, A and E_g, represents the absorption coefficient, planks constant, light frequency, proportionality constant related to the material, and energy band gap, respectively, and the value of n is 1 for TiO_2 and V_2O_5 with direct transition semiconductor properties [1].

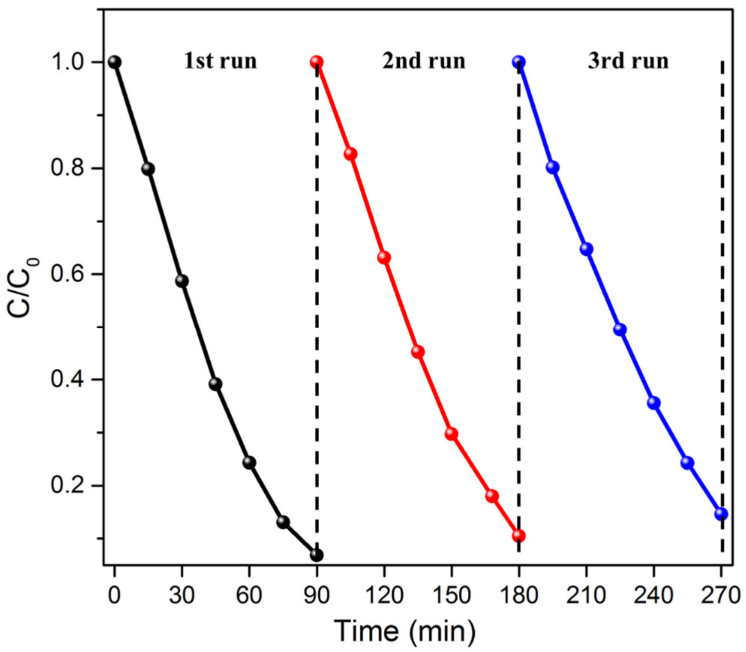

Figure S5. Recycling experiments of VOGTO2 photocatalyst for MB degradation.

The stability and reusability of prepared VOGTO2 photocatalyst were evaluated by a cycle experiment. Three successive recycling experiments were conducted by adding used VOGTO2 photocatalyst to fresh MB solution, all the used samples were collected after each cycle test, washed, and vacuum-dried at 80°C for 12 h. After Three cycles, no apparent deactivation of the photocatalyst is observed (a slight decrease derived from the loss of the photocatalyst during the cycling process), suggesting that the high stability of the VOGTO2 photocatalyst during the photodegradation of the MB (Figure S5).

References

1. Huang, K.; Hong, Y.; Yan, X.; Huang, C.; Chen, J.; Chen, M.; Shi, W.; Liu, C. Hydrothermal synthesis of g-$C_3N_4/CdWO_4$ nanocomposite and enhanced photocatalytic activity for tetracycline degradation under visible light. *CrystEngComm* **2016**, *18*, 6453–6463.
